# Assessing the Effectiveness of Local Management of Coral Reefs Using Expert Opinion and Spatial Bayesian Modeling

**DOI:** 10.1371/journal.pone.0135465

**Published:** 2015-08-18

**Authors:** Stephen S. Ban, Robert L. Pressey, Nicholas A. J. Graham

**Affiliations:** ARC Centre of Excellence for Coral Reef Studies, James Cook University, Townsville, Queensland, Australia; Leibniz Center for Tropical Marine Ecology, GERMANY

## Abstract

Multiple stressors are an increasing concern in the management and conservation of ecosystems, and have been identified as a key gap in research. Coral reefs are one example of an ecosystem where management of local stressors may be a way of mitigating or delaying the effects of climate change. Predicting how multiple stressors interact, particularly in a spatially explicit fashion, is a difficult challenge. Here we use a combination of an expert-elicited Bayesian network (BN) and spatial environmental data to examine how hypothetical scenarios of climate change and local management would result in different outcomes for coral reefs on the Great Barrier Reef (GBR), Australia. Parameterizing our BN using the mean responses from our experts resulted in predictions of limited efficacy of local management in combating the effects of climate change. However, there was considerable variability in expert responses and uncertainty was high. Many reefs within the central GBR appear to be at risk of further decline based on the pessimistic opinions of our expert pool. Further parameterization of the model as more data and knowledge become available could improve predictive power. Our approach serves as a starting point for subsequent work that can fine-tune parameters and explore uncertainties in predictions of responses to management.

## Introduction

In an ecological context, a stressor has been defined as a perturbation that is either foreign to the system being studied, or natural to that system but influencing it at an excessive level [1]. Multiple stressors are an increasing concern in the management and conservation of ecosystems because interactions between stressors can potentially exacerbate biodiversity declines [2–6]. Interactions between stressors can result in “ecological surprises” [7], as have been observed in freshwater [3], marine [8], and terrestrial [9] ecosystems. Multiple stressors do not affect ecosystems uniformly, however. Instead, they can exhibit spatial heterogeneity (e.g., salt marshes [10], and kelp beds [11]) that can affect the probability of regime shifts at particular sites [12].

Models of the effects of multiple stressors that provide spatially explicit outputs would be useful for informing management, yet few such models exist to date, at least in a marine context. Spatially explicit models would be particularly helpful in marine ecosystems, where ocean zoning has been proposed as one way of addressing cumulative impacts [13]. Interactions between stressors may occur at multiple spatial scales, ranging from local to global, with some stressors manageable, others not. Ecosystem managers (i.e., those responsible for recommending public policies directly or indirectly affecting an ecosystem) may be able to influence anthropogenic stressors by building resistance or resilience to non-manageable stressors such as storms and disease [14, 15]. With the increasing threat of climate change, assessing management options at the local and regional scales at which managers operate will be increasingly important [16, 17] as one way to delay or mitigate climate-change effects [8, 18, 19]. However, few practical approaches assess, model, or guide management of multiple stressors, particularly in marine ecosystems, largely due to data limitations. Data are limited partly by the number of interactions increasing exponentially with the number of stressors (i.e., the number of possible *n*-way interactions between *n* variables increases according to 2^n^), and by the general lack of ecological data from monitoring and assessment surveys that are sufficiently comprehensive to statistically examine the interactions between stressors. Additionally, estimating the strength of interactions between stressors is difficult for several reasons. One is the non-linear behaviour of ecosystem responses; another is the potential for mis-matches in timescale between discretely-measured empirical data and the assumption of instantaneous changes used by many mathematical models [20].

There are several modeling approaches for working in situations where data are either insufficient to fully parameterize a model, or provide insufficient statistical power for the number of variables being examined. Holmes and Johnstone [21], for example, used a dynamic systems model that was not spatially explicit but was parameterized at least partly with expert judgment and input. However, the predictive ability of models using classical inference is limited by scale-dependent effects, constraints on model dimension and parameterization, and uncertainty about which model components are stochastic or deterministic [22]. Some techniques that have been applied to multiple-stressor management have included qualitative ranking of ecosystem stressors by experts [23] and relative risk models [24]. Bayesian networks (BNs) have also found increasing application as a decision-support tool in ecology and adaptive management (e.g., [25], [26–28]) where predictive utility is paramount but data are limited and uncertainty is high [29]. Furthermore, the expert elicitation process commonly used in the development of Bayesian networks allows experts to contribute to the development of model structure as well as informing model parameters, thereby conceptualizing interactions between stressors as well as understanding their effects [25]. Expert elicitation has seen increasing use in decision-making in the context of problems in conservation biology where data are missing or incomplete [30].

Despite the increasing acceptance of BNs in ecology and management, spatial implementations of these models through management decisions remain rare; searching Web of Science using the keywords “Bayesian belief”, “spatial”, and “marine”, for example, finds only six papers [31–36]. In a marine-planning context, a recent paper [36] did use a BN in conjunction with a GIS to evaluate cumulative human impacts on the coastal waters of England and Wales; however, the cumulative impact score in Stelzenmüller et al.’s [36] study was simply the sum of the qualitative scores for each of three human activities given equal weighting, rather than a combination of stressors that recognized their different relative weights and effects.

In terms of evaluating ecosystem effects of climate change, many climate-change models and scenarios focus on slow-changing variables such as average temperature and acidification. Such models may not take into account interactions with variables that change on much shorter (annual or seasonal) timescales, and thus may underestimate the ecosystem effects of climate change. Thus few models are readily applicable to the short- and medium-term timeframes most useful for ecosystem managers (but see [37, 38]), and fewer still attempt to incorporate interaction effects between multiple stressors.

Multiple stressors have been identified as a key problem facing managers in coral reef ecosystems [16, 39] because reefs face threats on both global and local scales [40]. However, these threats vary over both time and space, and effective conservation planning for dynamic threats requires a spatially explicit prediction of those threats [41]. Furthermore, spatial heterogeneity becomes increasingly important for reserve system design as the scale of management diverges from the scale of underlying ecosystem processes [42]. Thus, in this paper, we spatially implement an expert-elicited Bayesian network (BN) in a coral reef ecosystem: the Great Barrier Reef (GBR) in Queensland, Australia. We chose the GBR as a case study because the key stressors (i.e., those having the greatest influence on reef condition) have been identified [43–49], and because good spatial data exist for many of these stressors. Stressors that may be locally manageable include nutrient loading, sedimentation, pollution, and fishing pressure, whereas stressors that are infeasible to manage directly include coral disease, coral bleaching, and cyclones. Outbreaks of crown-of-thorns (CoTS) starfish are possible to control, but the required management is highly labour-intensive and impractical at large scales [50]. Also, management of certain stressors, including CoTS, is made difficult by their unpredictability in time and space. Another advantage of the GBR as a case study is the existence of an extensive protected-area network, meaning that many reefs suffer little or no extractive pressures, allowing us to examine the potential effectiveness of these managed areas in reducing multiple-stressor effects, where coral cover is used as a proxy for reef condition.

Scenario planning is a way of considering possible futures for systems that contain high levels of uncertainty where direct experimental manipulations of the system are impractical [51]. Scenarios are one way to frame complex and uncertain factors that define the boundaries of a problem, but are neither forecasts nor predictions [52, 53]. In this study, we use scenarios in the form of possible combinations of stressor levels to model the effects of potential stressor interactions. We then use these model outputs to identify which management actions might most affect the future trajectory of coral cover. We present this study, not as a policy prescription, but as a first step towards better characterizing the complexity and realities of multiple stressors as they pertain to coral reefs. Our broad aim is to provide a foundation that can be built upon and fine-tuned to guide future management.

## Methods

### Ethics statement

Our survey design was approved by James Cook University’s Human Research Ethics Committee (Application ID #H4542). We obtained written consent from all survey participants.

### Model development

**Fig 1:**
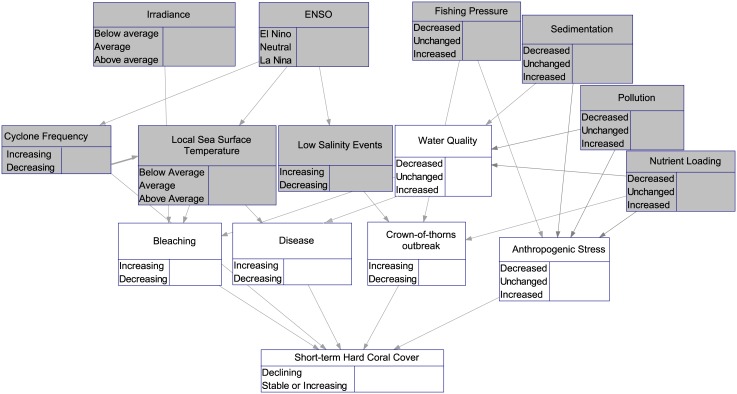
Schematic of the Bayesian network structure. Nodes in grey are informed by empirical data; nodes in white are elicited from experts. The nodes for water quality and anthropogenic stress are composite nodes that assign a weight to each of their parent nodes to create an overall index. Note that some of the nodes (e.g., bleaching, disease) reflect symptoms of stress, rather than being stressors *per se*.

We created a Bayesian network model using two types of data: empirical data and expert opinion (Fig 1). All of the spatial environmental data put into the model were empirical; the consequences of various combinations of these environmental conditions were generated from the BN model using expert opinions. Within our model, we devised a set of four scenarios, each one describing a different qualitative combination of possible conditions approximately 10 years into the future (Table 1). In these scenarios, changes in stressors or stressor indicators like bleaching and disease were described in terms of a one standard deviation change from the long-term mean in terms of either frequency for discrete events (cyclones, floods, crown-of-thorns outbreaks, mass bleaching, disease outbreaks) or intensity for fishing pressure, sedimentation, pollution, and nutrient loading. The choice of one standard deviation was arbitrary, but was chosen to be readily interpretable by the experts and consistent across variables, and to represent a significant enough change to lie outside the bounds of normal variations without being extreme enough to be unlikely. In the case of temperature, an increase of 1°C above the climatological mean was used as a conservative threshold value for coral bleaching [54]. It should be clarified that, in the survey, experts were asked how a temperature anomaly above this threshold would affect the chances of a mass bleaching event occurring, not the probability that these anomalies would occur. The scenarios were as follows:

1. Baseline: all variables unchanged from present conditions (i.e., at the time the surveys were conducted in 2012)
2. Climate change without local management: assume that temperature anomalies increase 0.2 degrees (based on 1°C of mean ocean warming by 2050) above those observed to date, with a concomitant increase in cyclone frequency, disease outbreaks, and mass bleaching events, without any reductions in fishing pressure or terrestrial inputs (nutrients, sediments, pollution)
3. Climate change with local management: as in the previous scenario, but with management actions to reduce fishing pressure and terrestrial inputs (i.e. terrigenous nutrient, sediment, pollutants) by approximately one standard deviation (~30%) relative to current levels.
4. Local management without further climate change: implementation of management actions as in the previous scenario, but without any change in climate-related variables beyond present conditions.

**Table 1:** Climate change and management scenarios with associated changes to input layers. n.c. = no change from baseline (current conditions); plus sign = 1 standard deviation increase above baseline; minus sign = 1 standard deviation decrease below baseline condition, except for temperature (0.2 degree above/below climatological mean). CoTS = Crown-of-thorns-starfish. Scenarios refer to the change in conditions after approximately a 10-year timeframe.

Scenario number (see text for description)	Temperature	Cyclones	Disease	Bleaching	Irradiance	CoTS	Nutrients	Sediment	Pollution	Fishing
1	n.c.	n.c.	n.c.	n.c.	n.c.	n.c.	n.c.	n.c.	n.c.	n.c.
2	+	+	+	+	n.c.	n.c.	n.c.	n.c.	n.c.	n.c.
3	+	+	+	+	n.c.	n.c	-	-	-	-
4	n.c.	n.c.	n.c.	n.c.	n.c.	n.c.	-	-	-	-

The empirical data for the model came from several sources (Table A in S1 File, grey nodes in Fig 1). Historical cyclone tracks were obtained from IBTRACS [55]. Only Category II and higher cyclones were included, and the tracks were buffered asymmetrically as per Fabricius et al. [56]. The average and maximum extent of flood plumes from 2007–2011 were obtained from Alvarez-Romero et al. [57], as were data on loadings of nutrients (dissolved inorganic nitrogen) and sediment. Sea surface temperature anomalies were identified from satellite climatology data (1985–2007) as per the methods described in Ban et al. [58]. Mean solar irradiance was obtained from NASA SeaWiFS satellite data (1997–2010). Data on fishing catch and effort for line fisheries were obtained from the Queensland Department of Agriculture, Fisheries and Forestry. Some data on commercial catch and effort were available only at a resolution of 30 nautical miles (nm) due to confidentiality rules, whereas others had a resolution of 6 nm. We downscaled the lower-resolution data by assuming that the relative effort distribution of the data at 30 nm resolution was similar to that of the 6 nm-resolution data, and reapportioned the 30 nm data accordingly. This approach is commonly used in stock assessments of Queensland fisheries [59]. Reefs within no-take areas were assumed to have no fishing effort or catch. Except for temperature, all input data layers were re-coded into three categories to be consistent with the survey questions, which specified 3 possible states or conditions for each variable: 1 standard deviation or more above average; within 1 standard deviation of average; and 1 standard deviation or more below average. For temperature, anomalies were coded as being greater than/less than/within 1°C from the climatological mean. All of the input layers, consisting of spatially-explicit values for individual reefs, were then placed in different combinations for each of our scenarios (Table 1).

The second type of data used in the model was based on expert opinions about the degree of influence each empirical variable had on the probability of a certain event (white nodes, Fig 1). The expert pool consisted of 21 coral reef ecologists with extensive experience with the GBR specifically. These experts were asked to consider the probability of various events occurring within a ten-year timeframe for a hypothetical mid-shelf, mid-latitude reef with approximately 30% hard coral cover. Experts were interviewed on an individual basis, using the 4-point elicitation method [60], whereby respondents are asked for the lowest possible value, highest possible value, best estimate, and confidence in their estimate. Prior to answering the questionnaire, to reduce variability, each expert was read a statement that provided definitions for key terms and set the context of the questions. Experts were also provided with a schematic diagram of the model, and asked to annotate the diagram to add or delete any stressors or stressor relationships as necessary. The initial selection of the expert pool—based on scientists with the largest number of publications pertaining to coral reef ecology on the GBR—was expanded using the snowball method [61], wherein each expert was also asked to provide the names of two other people whom they recognized as experts in the field. In our model, the events considered by the experts were: probability of a mass bleaching event, probability of a coral disease outbreak, and probability of a crown-of-thorns starfish (CoTS) outbreak. For nodes where the number of possible permutations of event states was impractical to elicit directly from our experts, we instead asked for probability estimates of the extreme endpoints (all stressors high vs. all stressors low), and interpolated between those estimates. The complete methodology of this expert elicitation process is described in Ban et al [62]. The ultimate endpoint of the model was the probability of hard coral cover declining below present levels over a ten-year timeframe (2012–2022), which was also estimated by our experts. This probability was contingent upon the various events (bleaching, disease, CoTS) increasing or decreasing in frequency. We used three sets of probabilities of events from the expert elicitation process as inputs (parameterizations) to the model: the group mean, the 25^th^ percentile (pessimistic), and the 75^th^ percentile (optimistic). The pessimistic parameterization corresponded to higher probabilities of adverse events; the optimistic parameterization corresponded to lower probabilities of adverse events.

Both the empirical data and the expert-elicited probabilities were entered in the form of conditional probability tables into Netica [63], where each unique combination of event states (e.g, high/average/low temperature, presence/absence of a flood plume) is associated with a discrete probability of an outcome (e.g., mass bleaching). All data layers were plotted onto a common 500 metre geographic grid, which was the finest resolution of the input layers. Model predictions for the probability of coral cover declining below current levels were then calculated for each mid-shelf reef by supplying the model with input values from that location. These overall probabilities of decline were also the result of expert opinions regarding various combinations of events (e.g., a mass bleaching event and a disease outbreak event occurring in close succession on the same reef). Additionally, due to the decade-scale time frame, these probabilities of decline were intended to capture the net result of both mortality events and subsequent recovery (i.e., whether a reef would be able to recover its pre-disturbance coral cover following a certain combination of events).

**Fig 2:**
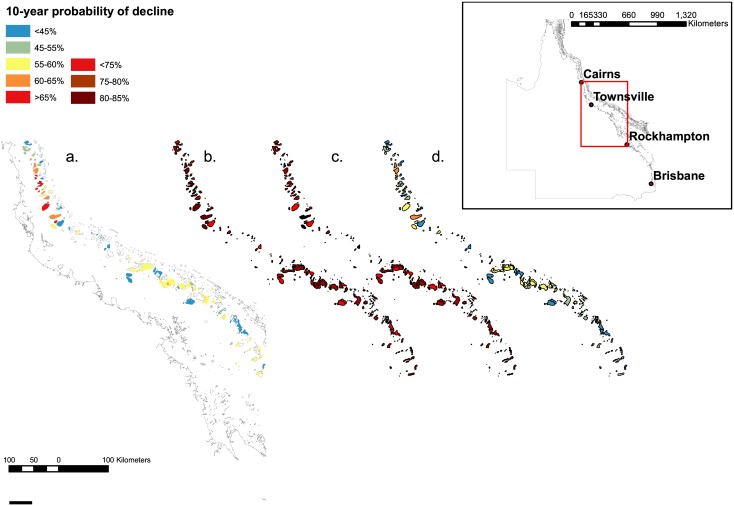
Model outputs for 10-year probability of decline of hard coral cover for each scenario: a) baseline; b) climate change without local management c) climate change with local management; d) local management without further climate change. The baseline scenario map (a) depicts all reefs, including inner- and outer-shelf reefs for context; model results were applied only to mid-shelf reefs. Inset: study area, focused on central portion of the GBR, excluding far-northern and far-southern reefs.

We limited the extent of the study area for two reasons: first, to match the availability of our input spatial data layers, and second to avoid over-generalizing the applicability of our scenarios given the very different characteristics of inshore, mid-shelf, and offshore reefs. Our study area was thus confined to the central GBR (inset, Fig 2). We constrained the model inputs and outputs only to those areas designated as “mid-shelf” under GBRMPA’s bioregional classification system [64, 65]; this encompassed 775 individual reefs in a latitudinal range extending between 15.77°S and 22.31°S, corresponding with the minimum extent of the data input layers.

The four scenarios compared model predictions using four different combinations of conditions: with and without management action, and with and without additional climate change effects (Table 1; Table B in S1 File). These four scenarios were: Baseline, Climate Change without Local Management, Climate Change with Local Management, and a best-case scenario of Management without (further) Climate Change. The Baseline scenario represented the mean of the available environmental data for each grid cell. Under the climate-change scenarios, we increased the risk category uniformly for cyclones so that the chance of any given reef being hit by a cyclone increased by 30%, added 0.2°C to historical temperature anomalies (based on the IPCC A1B scenario of ~2.2°C average SST rise by 2100; [66]), and increased the extent/severity of flood plumes, sedimentation, and nutrient loading by one standard deviation (~30%). Incident irradiance and frequency of CoTS outbreaks remained unchanged across all scenarios. In the improved management scenarios, fishing pressure, sedimentation, and nutrient loading were reduced by one standard deviation.

We used the centroid (geographic centre) of each reef within the study area to determine the values of the input layers, and mapped the corresponding model-predicted probability of coral decline for each scenario at that location. The output values of the model thus corresponded to the input values for the centroid of each reef. We then compared the average probability of predicted decline of hard coral cover for reefs both within highly protected (no-take) zones (green, orange, and pink zones: [67]) and outside these zones. Finally, we produced a series of change maps by taking the difference in predicted probability of decline between two scenarios and calculating the relative change by dividing this difference by the first scenario’s predicted value.

1. Climate change with local management vs. baseline
2. Climate change with local management vs. climate change without local management
3. Local management without further climate change (best-case scenario) vs. baseline

## Results

Under the baseline scenario, our model predicted probabilities of decline for mid-shelf reefs ranging from about 35% to 75% (Fig 2a), with a mean of 59% with respect to the number of reefs. Under the climate change without local management scenario, few reefs had a probability of decline of less than 70%, with some having probabilities of more than 85% and most falling into the 70–80% range (Fig 2b). The mean probability of decline in this scenario was 77%. Under the climate change with local management scenario, in which fishing pressure, sedimentation, and nutrient loading were reduced by at least 30% (Fig 2c), the mean decline was also 77%, and most reefs showed a very similar probability of decline to those in the scenario of climate change without local management. Finally, under the best-case scenario (reduced fishing, sediment and nutrient loading without further climate change), the mean predicted probability of decline was 58% (Fig 2d). In short, the baseline scenario resulted in a mean probability of decline of 59%, climate change with or without management action had a probability of decline of 77%, and the best-case scenario had a mean probability of decline of 58%.

**Fig 3:**
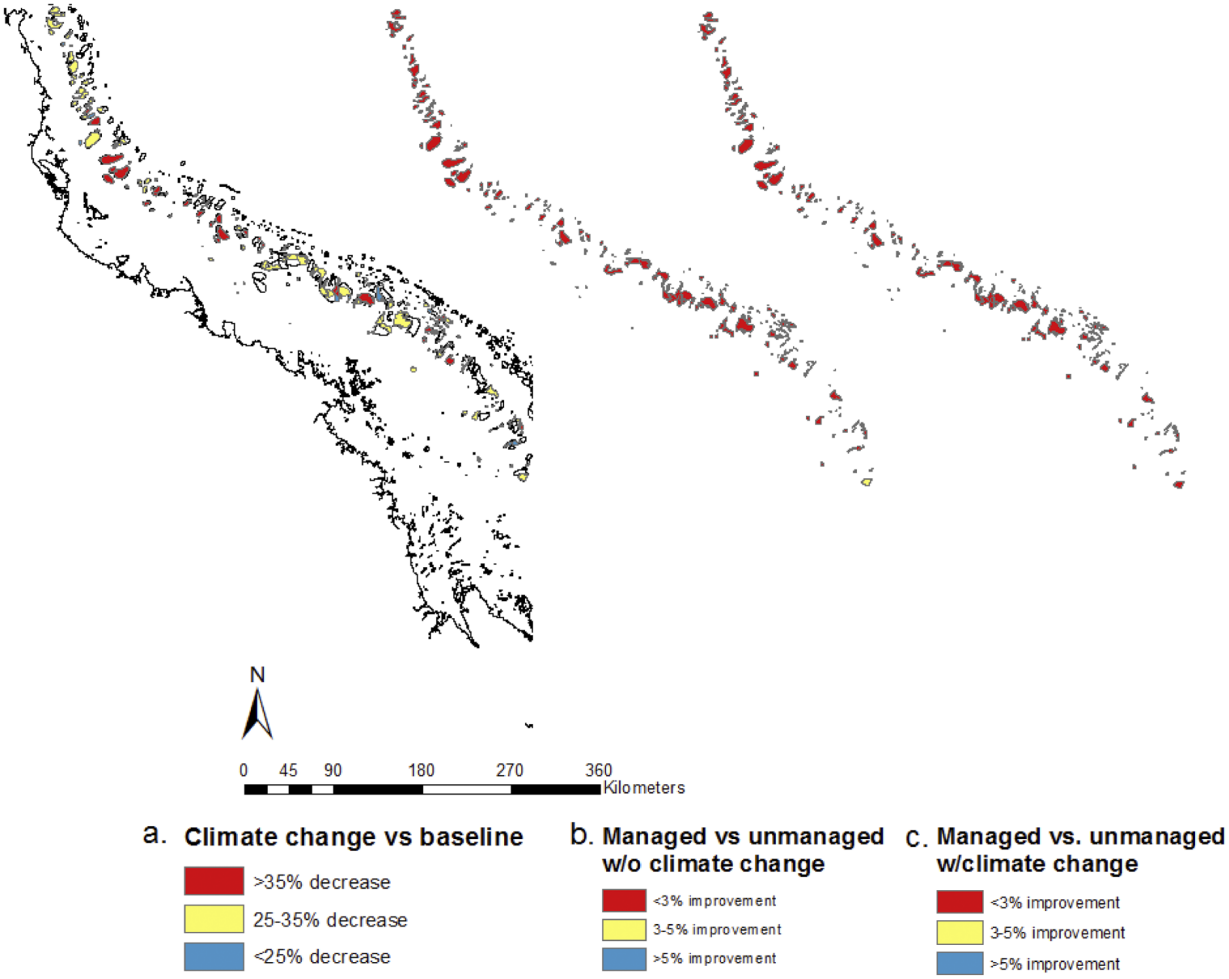
Relative change in probability of decline in hard coral cover between two scenarios: climate change without local management and the baseline scenario. Differences between other scenarios not shown because they were minimal.

**Fig 4:**
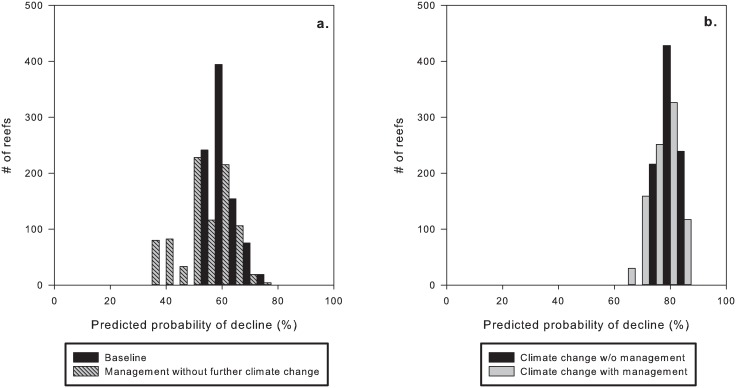
Difference in decline probability between paired management scenarios. a) Baseline vs management without further climate change. b) Climate change with management action vs climate change without management action.

The climate change without local management scenario increased the mean probability of decline by approximately 30% over the baseline value, from a mean decline probability of 59% to 77% (Fig 3). For the other scenario pairs, although the means differed very little, the distributions of decline probabilities did differ (Fig 4). Local management without further climate change (best-case) reduced the probability of decline by a mean of 0.7% relative to baseline (Fig 4a). Climate change with local management resulted in a mean 0.3% reduction of probability of decline relative to climate change without local management (Fig 4b).

**Fig 5:**
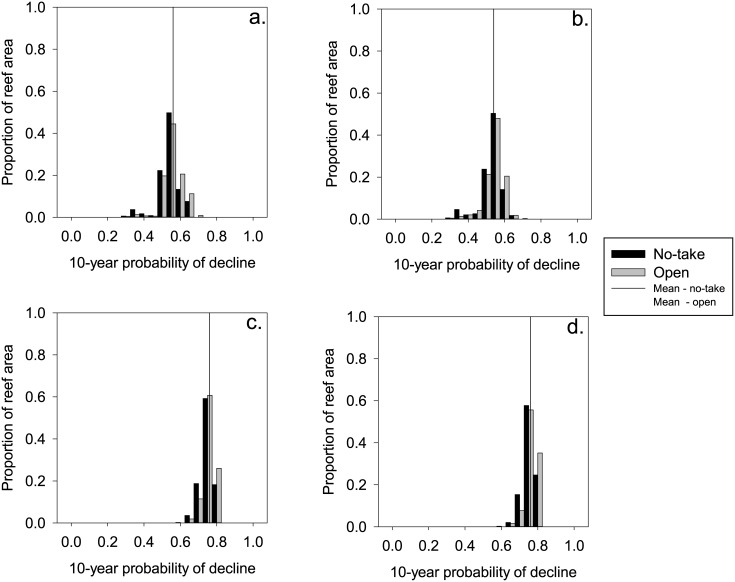
Comparison of predicted 10-year decline probabilities between all four scenarios: a) baseline vs management with no further climate change; b) climate change with management action vs climate change without management action.

Reefs outside no-take zones had marginally higher mean probabilities of decline than reefs inside no-take zones in each of our scenarios (Fig 5). The mean difference in probability of decline between no-take and other zones ranged from just under 1% in the climate change with local management scenario (Fig 5d) to just over 5% in the baseline scenario (Fig 5a).

Using the 25^th^ (pessimistic) and 75^th^ (optimistic) percentile model parameterizations with the same input data scenarios shifted the distribution of probabilities of decline accordingly (Figs A,B in S1 File). There was less variability between reefs in both the 25^th^ and 75^th^ percentile parameterizations compared to the mean parameterization, particularly in the case of the 25^th^ percentile under both climate change scenarios (Fig B in S1 File), for which the mean predicted probability of decline was more than 90%.

## Discussion

We used an expert-elicited BN to explore possible differences in vulnerability to multiple stressors on mid-shelf reefs within the GBR, and found a moderate probability of continued decline in coral cover, even in the best-case scenario of no further climate change combined with reductions in local stressors. Our intent was to construct a model that could be used to generate spatially explicit outputs using only basic and readily-available input data and to compare hypothetical outcomes of different management and stressor scenarios over a relatively short (10-year) time frame. However, given the relatively coarse parameterization and applicability of the models, and the range of likely values in the expert responses (see Ban et al [62] for more detail on the spread of parameter values), we believe this should be only the first step in constructing more sophisticated models that are tailored specifically to different types of reefs.

Our results highlight experts’ opinions of the dire situation of coral reefs in the GBR, as well as the uncertainty or lack of consensus between experts about many stressor effects. Even under our baseline scenario, in which we assumed that all stressors remained unchanged, our model predicted a moderate probability that coral cover on mid-shelf reefs would decline. This result is consistent with recent findings that coral cover on the Great Barrier Reef as a whole has been declining at an average rate of 0.5% per year since surveys began in 1985, with a steepening rate of decline since 2006 [44]. Perhaps more surprising is how little the best-case scenario (reduction of all locally-manageable stressors without further climate change) differed from the baseline scenario. There are several possible explanations for this lack of change. One is that many mid-shelf reefs rarely experience the effects of terrigenous flood plumes and their associated chemicals, sediment, and nutrients [68], so reductions in any or all of these factors would be unlikely to have a significant effect on the trajectory of coral cover on these reefs. Secondly, the effects of stressors that are not under direct management control (such as outbreaks of CoTS, mass bleaching, and cyclones) are likely to have a much stronger immediate influence on coral mortality and subsequent declines in cover than many of the manageable stressors. This also comports with the findings of De’ath et al [44], who concluded that CoTS outbreaks and cyclones, along with bleaching, were key drivers of coral decline in the GBR.

Based on expert opinion, our model predicted a slight but consistent difference in the predicted probability of decline between reefs within and outside the existing protected area network on the GBR. Since fishing pressure is one of the stressors in the model, and we assumed that fishing pressure inside protected areas was zero, this result is not surprising. Research on the GBR has shown higher coral cover on reefs inside versus outside no-take areas—partly due to protection from direct damage from fishing gear and anchors and partly from indirect effects possibly associated with trophic interactions [69]. More generally, fishing pressure has been linked to changes in coral cover through a trophic cascade process [70, 71]. Although weak, the association between fishing pressure and probability of declining coral cover in our model remaining even in all of the improved management scenarios where the 30% reduction in fishing pressure was simulated to occur outside reserves. While commercial fishing pressure within the GBR is thought to be sustainable [72], insufficient data exist for many species, and increased shark landings are a particular concern [73]; thus, a 30% across-the-board reduction in fishing may be less effective than larger catch reductions in selected keystone or apex species [74, 75]. Since our model did not incorporate patterns of larval dispersal or connectivity between reefs, it did not capture the benefits of a protected area network as a recruitment source, and overlooked some of the other benefits of marine reserves beyond direct fishing impacts, such as trophic cascade effects that may reduce outbreaks of crown-of-thorns starfish and sea urchins and so result in increases in coral cover [14, 69, 76].

Our findings should not be interpreted to mean that local management actions to mitigate or reverse declines in coral cover are futile or unimportant. Recent studies have demonstrated possible effects of local management on enhancing resistance and resilience of coral reefs to stresses [14, 17, 18, 77]. One example is where rebuilding fish biomass and diversity may increase the resistance of reefs to disturbances such as bleaching and disease [77]. The effectiveness of local management actions may also depend on the nature of the interaction between stressors, because reducing a stressor that is interacting in an antagonistic or mitigative fashion with another may actually make the net result worse [18, 78]. However, marine reserves are not a panacea for dealing with stress and disturbance to coral reefs [79–83], particularly if activities other than fishing are not restricted. Other local management actions include reductions in sediment and pollutant loading. One possible implication of our model is that the current level of anthropogenic stress is such that even a 30% reduction in all local impacts may be insufficient to stop or reverse the trend of declining coral cover, and thus that more drastic management interventions are required. For example, Wooldridge et al [84] reported that a 50–80% in dissolved inorganic nitrogen inputs would be necessary to return the GBR to pre-European conditions.

There are some limitations to the model we have developed. First, it was parameterized for only one type of reef (mid-shelf and mid-latitude) within the GBR, and it assumes uniform susceptibilities to threats like disease and bleaching, regardless of community composition. Since our model was high-level and not mechanistic, it can only paint a general picture of these reefs’ possible responses to stressor scenarios. We also deliberately confined the model to have a limited number of input states. This was mainly a limitation of the expert-elicitation approach and the need to have a tractable number of stressor permutations within scenarios, but it also required us to interpolate responses of intermediate stressor conditions linearly between endpoints (See [62] for more details). It is also important to recognize the limitations of expert judgment, especially in tasks requiring weighting and combining factors [85]. Our model was also intended as a form of hypothesis generation and testing rather than as a prescriptive guide. Furthermore, conclusions about the effectiveness of management actions depend upon the structure of the model; different model structures would likely result in quite different qualitative (and quantitative) predictions.

Our study is one of the few examples of expert-elicited Bayesian networks that have been applied spatially in a marine environment [31–36]. Expert elicitation has seen increasing application in ecological contexts where empirical data are absent, incomplete, or uncertain and is especially useful in combination with Bayesian methods [86]. While the model we developed and applied here was somewhat simplified, it is also one of the few that has attempted to capture the effects of simultaneous multiple stressors in an explicit fashion where the nature of stressor interactions (e.g., additive, synergistic, mitigative) is not assumed a priori. Despite their relatively crude nature, coarse or approximate models can still be useful as a decision-support tools [87], and conservation biology in particular benefits from the use of such models to “simulate uncertain but plausible scenarios, explore the consequences of decisions, and test alternative decision strategies” [87]. Further development of this model—such as by developing more sophisticated sub-models and incorporating specific elements affecting recovery and resilience factors, and, if possible, ground-truthing the results—will likely be necessary before it can be used in a real-world management context. However, spatially-explicit models informed by expert-opinion are ultimately likely to be useful to assess and prioritize areas of conservation concern in many data-limited and/or time-sensitive systems beyond coral reefs. Such models should, of course, also be subjected to peer-review before being applied in a management context.

## Supporting Information

S1 File Input data layers (Table A). Modified environmental data layers for scenarios. (Table B) Comparison between optimistic, pessimistic, and mean model outputs for probability of decline of hard coral cover for a) baseline scenario, reefs open to fishing; b) baseline scenario, reefs closed to fishing; c) Local management without further climate change, open reefs; and d) Local management without further climate change, closed reefs. (Fig A) Comparison between optimistic, pessimistic, and mean model outputs for probability of decline of hard coral cover for a) climate change without local management scenario, reefs open to fishing; b) climate change without local management, reefs closed to fishing; c) climate change with local management, open reefs; and d) climate change with local management, closed reefs. (Fig B).
(DOCX)
