# Supplementary material for: Assessing the Effectiveness of Local Management of Coral Reefs Using Expert Opinion and Spatial Bayesian Modeling
Source: PLoS One. 2015 Aug 18;10(8):e0135465. doi: 10.1371/journal.pone.0135465 (PMC4540441; doi:10.1371/journal.pone.0135465)
Supplement: S1 File — Input data layers (Table A). Modified environmental data layers for scenarios. (Table B) Comparison between optimistic, pessimistic, and mean model outputs for probability of decline of hard coral cover for a) baseline scenario, reefs open to fishing; b) baseline scenario, reefs closed to fishing; c) Local management without further climate change, open reefs; and d) Local management without further climate change, closed reefs. (Fig A) Comparison between optimistic, pessimistic, and mean model outputs for probability of decline of hard coral cover for a) climate change without local management scenario, reefs open to fishing; b) climate change without local management, reefs closed to fishing; c) climate change with local management, open reefs; and d) climate change with local management, closed reefs. (Fig B). (DOCX) [file pone.0135465.s001.docx]

S1 Table A. Input data layers, associated spatial and temporal resolutions, and data sources.

| Layer | Spatial Resolution | Temporal resolution | Source | Starting date | Ending date | Notes |
| --- | --- | --- | --- | --- | --- | --- |
| Irradiance | 9km^2^ | 1 month | SeaWIFS/AQUA | Sep-1997 | Dec-2010 | AQUA data used for temporal gap-filling |
| ENSO | Non-spatial | 1 month | Australian Bureau of Meteorology | Jan-1906 | Sep-2012 |  |
| Cyclone frequency | Non-spatial | 1 day | Australian Bureau of Meteorology | Jan-1906 | Jan-2012 |  |
| Cyclone tracks | n/a | 1 day | IBTRACS – International Best Tracks Archive for Climate Stewardship ([Knapp et al. 2010](#_ENREF_3)) | Jan-1885 | Dec-2008 | >Cat 2 cyclone tracks, buffered 50km to the left and 30km to the right |
| Flood events | River basin | 1 day | Australian Bureau of Meteorology | Apr-1901 | Sep-2012 |  |
| Flood plume extent | 0.5km^2^ | Yearly | ([Alvarez-Romero et al. 2013](#_ENREF_1)) | 2007 | 2011 |  |
| Sea surface temperature | 4km^2^ | Monthly | NOAA Pathfinder/([Ban et al. 2012](#_ENREF_2)) | Jan-1985 | Dec-2009 |  |
| Sedimentation | n/a | Yearly | ([Alvarez-Romero et al. 2013](#_ENREF_1)) | 2007 | 2011 |  |
| Nutrient loading | 0.5km^2^ | Yearly | ([Alvarez-Romero et al. 2013](#_ENREF_1)) | 2007 | 2011 | DIN only |
| Pollution |  | Yearly | ([Maughan et al. 2008](#_ENREF_4)) | Modeled based on data up to 2006 | Modeled based on data up to 2006 | Herbicide (primarily diuron) loading only |
| Commercial fishing catch/effort | 6nm^2^ (~11km^2^) | Yearly | Queensland Department of Agriculture, Forests, and Fisheries | 2001 | 2012 | Only line-caught species included; trawls and nets excluded |

S1 Table B. Modified environmental data layers for scenarios

|  | Sea surface temperature | Irradiance | Cyclone tracks | Flood plume extent | Sedimentation | Nutrients | Pollution | Fishing |
| --- | --- | --- | --- | --- | --- | --- | --- | --- |
| Baseline | As is: Average 1-degree anomalies; 3-standard deviation cutoff | As-is: 1 SD climatology anomalies | Uniform average risk category | Average extent across 2007-2011 | Averaged across 2007-2011 | Averaged across 2007-2011 | Averaged across 2007-2011 | As-is; zero fishing assumed inside reserves |
| No climate change with local management | Same as baseline | Same as baseline | Uniform average risk category | 30% decrease in flood plume extent | 30% decrease in sedimentation extent | 30% decrease in sedimentation extent | 30% decrease in sedimentation extent | 30% decrease in sedimentation extent |
| Climate change with local management | +0.2C to summer anomalies | Same as baseline | Uniform increased risk category | 30% increase in flood plume extent | 30% decrease in sedimentation extent | 30% decrease in sedimentation extent | 30% decrease in sedimentation extent | 30% decrease in sedimentation extent |
| Climate change without local management | +0.2C to summer anomalies | Same as baseline | Uniform increased risk category | 30% increase in flood plume extent | 30% increase in sedimentation extent | Same as baseline | Same as baseline | Same as baseline |

S1 Fig. A. Distribution of probabilities of decline in hard coral cover in relation to proportion of total reef area for models using 75^th^ percentile (optimistic), 25^th^ percentile (pessimistic), and mean expert parameters for: a) baseline scenario, reefs open to fishing; b) baseline scenario, no-take areas; c) local management without further climate change, open reefs; and d) local management without further climate change, no-take reefs. Dotted vertical line shows mean for 75^th^ percentile estimates; solid vertical line shows mean for average estimates; dashed vertical line shows mean for 25^th^ percentile estimates.


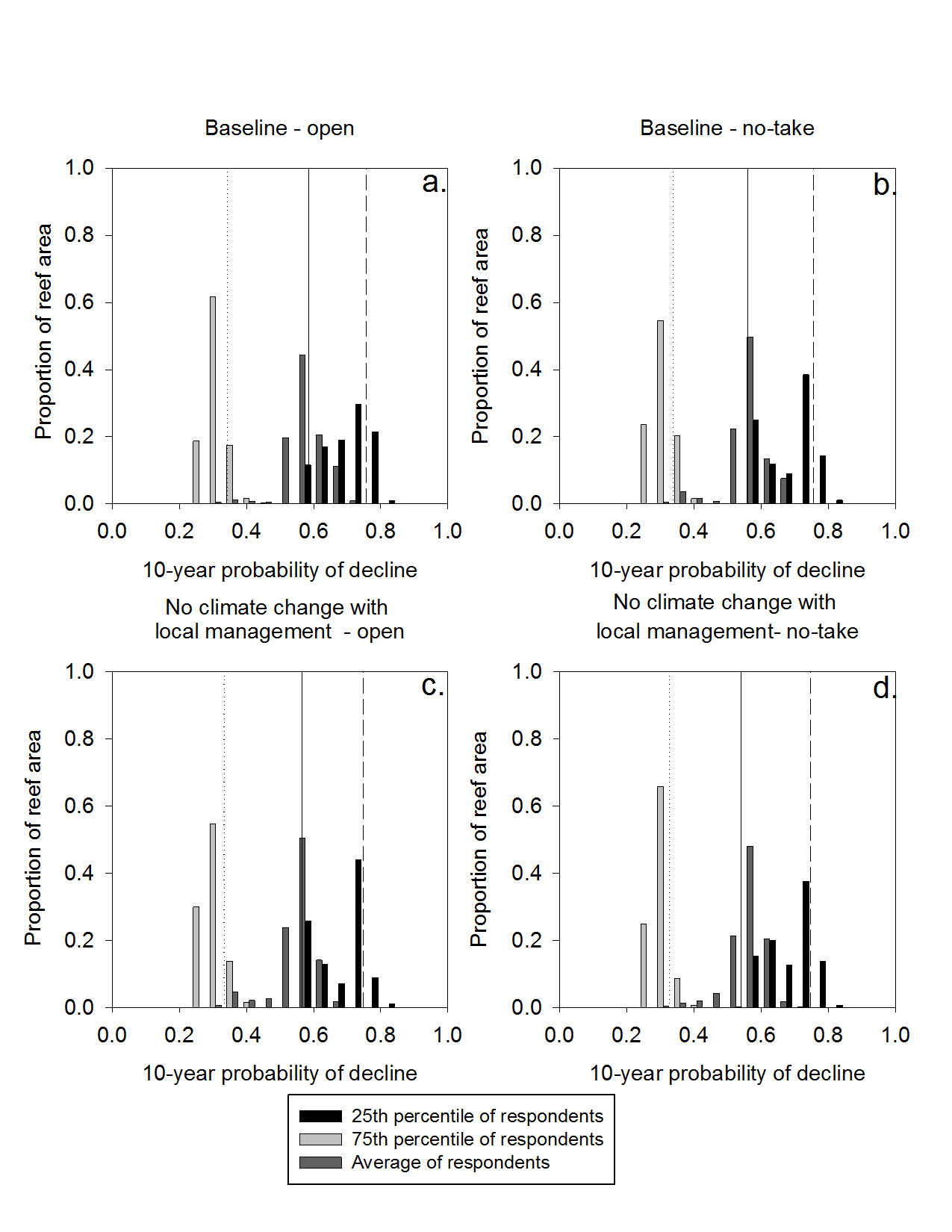


S1 Fig. B. Distribution of probabilities of decline in hard coral cover by reef area for models using 75^th^ percentile (optimistic), 25^th^ percentile (pessimistic), and average expert parameters for: a) climate change without local management scenario, reefs open to fishing; b) climate change without local management, no-take reefs; c) climate change with local management, open reefs; and d) climate change with local management, no-take reefs. Dotted vertical line shows mean for 75^th^ percentile estimates; solid vertical line shows mean for average estimates; dashed vertical line shows mean for 25^th^ percentile estimates.


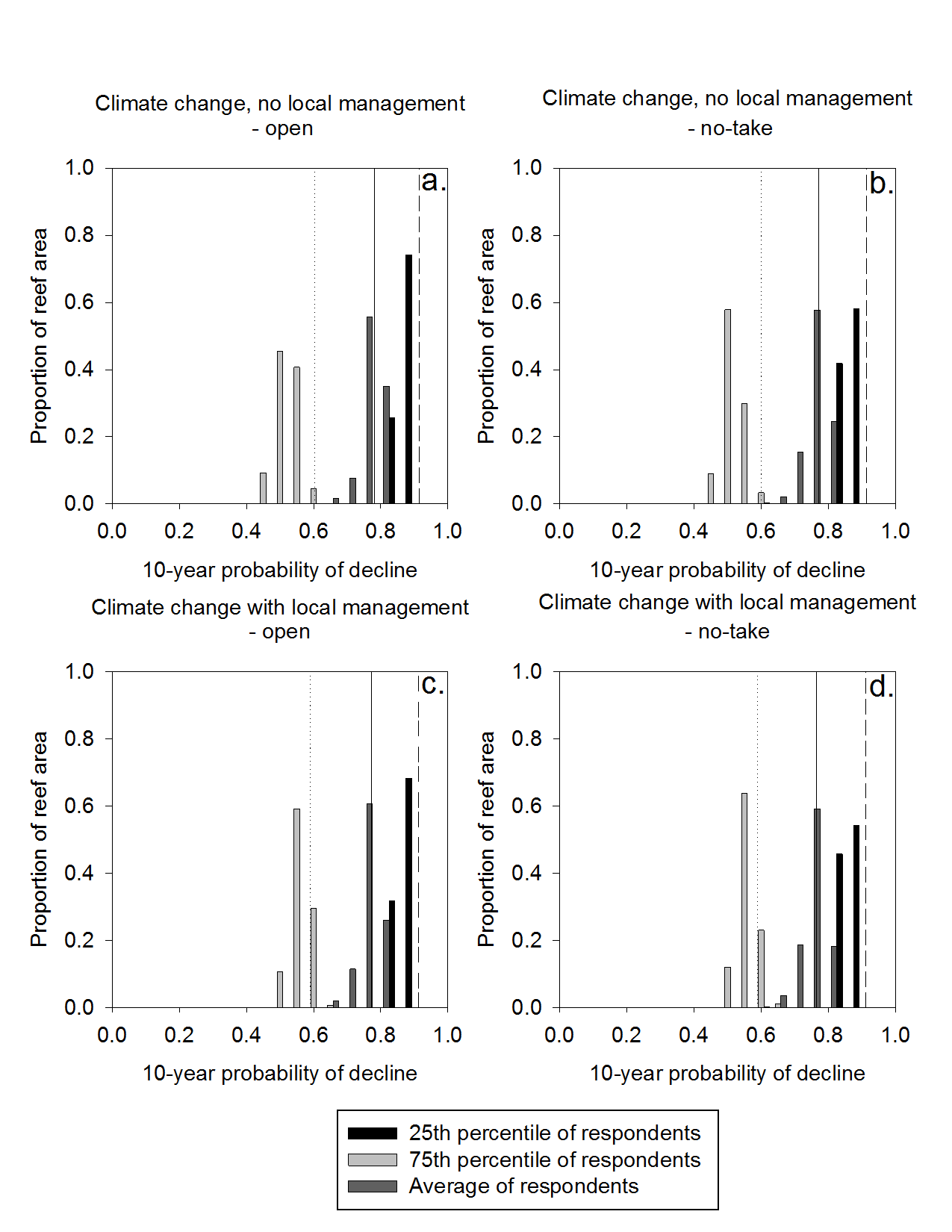


**References**

Alvarez-Romero J.G., Devlin M., Petus C. *et al.* (2013) A novel approach to model exposure of coastal-marine ecosystems to riverine flood plumes based on remote sensing techniques. *Journal of Environmental Management* **119**, 194-207.

Ban N.C., Pressey R.L., Weeks S. (2012) Conservation objectives and sea-surface temperature anomalies in the Great Barrier Reef. *Conservation Biology* **26**, 799-809.

Knapp K.R., Kruk M.C., Levinson D.H., Diamond H.J., Neumann C.J. (2010) The international best track archive for climate stewardship (IBTrACS): Unifying tropical cyclone best track data. *Bulletin of the American Meteorological Society* **91**, 363-376.

Maughan M., Brodie J., Waterhouse J. (2008) Reef exposure model for the Great Barrier Reef lagoon. p. 153. Australian Centre for Tropical Freshwater Research.
